# The Diet–Obesity–Brain Axis: Metabolic, Epigenetic, and DNA-Repair Pathways Linking Eating Patterns to Cognitive Aging, with an AI-Enabled Translational Perspective

**DOI:** 10.3390/nu17213493

**Published:** 2025-11-06

**Authors:** Manish Loomba, Sanjeev Bansal, Krishna Kumar Singh, Pradeep Kumar Mishra, Shampa Ghosh, Manchala Raghunath, Awdhesh Kumar Mishra, Jitendra Kumar Sinha

**Affiliations:** 1 Amity University Uttar Pradesh, Sector 125, Noida 201303, Uttar Pradesh, India; 2 Symbiosis Centre for Information Technology, Symbiosis International (Deemed University), Rajiv Gandhi InfoTech Park, Hinjawadi, Pune 411057, Maharashtra, India; 3 TCG Centres of Research and Education in Science and Technology, Bengal Eco Intelligent Park, Sector-V, Salt Lake, Kolkata 700091, West Bengal, India; 4 GloNeuro, Sector 107, Vishwakarma Road, Noida 201301, Uttar Pradesh, India; 5 ICMR—National Institute of Nutrition (NIN), Tarnaka 500007, Hyderabad, India; 6 Department of Biotechnology, Yeungnam University, Gyeongsan 38541, Republic of Korea

**Keywords:** artificial intelligence, Mediterranean diet, MIND diet, obesity, neuroinflammation, oxidative stress, DNA repair, NAD^+^, microbiome-gut-brain axis

## Abstract

Diet influences brain health through many connected metabolic and molecular pathways, and these effects are stronger in obesity. This review links diet quality with cognitive decline and dementia risk. Ultra-processed, high-fat, high-sugar diets drive weight gain, insulin resistance, and chronic inflammation. These changes trigger brain oxidative stress, reduce DNA repair, deplete NAD^+^, disturb sirtuin/PARP balance, and alter epigenetic marks. Gut dysbiosis and leaky gut add inflammatory signals, weaken the blood–brain barrier, and disrupt microglia. Mediterranean and MIND diets, rich in plants, fiber, polyphenols, and omega-3 fats, slow cognitive decline and lower dementia risk. Trials show extra benefit when diet improves alongside exercise and vascular risk control. Specific nutrients can help in certain settings. DHA and EPA support brain health in people with low omega-3 status or early disease. B-vitamins slow brain shrinkage in mild cognitive impairment when homocysteine is high. Vitamin D correction is beneficial when levels are low. A practical plan emphasizes healthy eating and good metabolic control. It includes screening for deficiencies and supporting the microbiome with fiber and fermented foods. Mechanism-based add-ons, such as NAD^+^ boosters, deserve testing in lifestyle-focused trials. Together, these measures may reduce diet-related brain risk across the life span. At the same time, artificial intelligence can integrate diet exposures, adiposity, metabolic markers, multi-omics, neuroimaging, and digital phenotyping. This can identify high-risk phenotypes, refine causal links along the diet–obesity–brain axis, and personalize nutrition-plus-lifestyle interventions. It can also highlight safety, equity, and privacy considerations. Translationally, a pattern-first strategy can support early screening and personalized risk reduction by integrating diet quality, adiposity, vascular risk, micronutrient status, and microbiome-responsive behaviors. AI can aid measurement and risk stratification when developed with privacy, equity, and interpretability safeguards, but clinical decisions should remain mechanism-aligned and trial-anchored.

## 1. Introduction

Diet is a powerful and modifiable determinant of brain health throughout life [1]. The brain has high metabolic demand, rich lipid composition, and limited antioxidant reserves. These features make it vulnerable to diet-driven metabolic stress. Chronic positive energy balance, hyperglycemia, and dyslipidemia are key stressors. They damage brain metabolism and function. Excess adiposity adds a second hit. It drives low-grade, systemic inflammation. This inflammation reaches the central nervous system (CNS) and worsens brain vulnerability [2]. Pathways include blood–brain barrier (BBB) disruption, microglial activation, bioactive lipid signaling, and altered insulin/leptin signaling. This metaflammation spreads beyond hypothalamic circuits controlling energy balance. It also affects the hippocampus, cortex, amygdala, and brainstem, regions critical for memory, executive function, and mood [3]. At the cellular level, oxidative stress and impaired DNA damage responses connect diet and obesity to neuronal vulnerability. Reactive oxygen species (ROS) increase with overnutrition, mitochondrial overload, and chronic inflammation [4]. In neurons, sustained ROS drives lipid peroxidation, protein oxidation, and DNA lesions. These changes impair synaptic function and bioenergetics [5]. Current evidence identifies oxidative stress as a core driver of brain aging and neurodegeneration. It links mitochondrial dysfunction and redox imbalance to cognitive decline [6,7].

DNA integrity is especially important in long-lived, post-mitotic neurons. When oxidative or metabolic stress increases DNA damage, neurons activate repair programs [8]. These programs are energetically costly and can disturb cellular homeostasis if chronically engaged. Reviews agree that cumulative DNA damage and deficits in repair pathways speed neuronal aging and raise neurodegeneration risk [9]. NAD^+^ metabolism forms a unifying biochemical axis for these processes. NAD^+^ acts as both a redox cofactor and a substrate for sirtuins and PARPs [10]. These enzymes regulate stress responses, DNA repair, mitochondrial function, and chromatin structure. Age- and stress-related NAD^+^ depletion can reduce DNA repair efficiency and weaken sirtuin signaling [11]. This loss may lower neuronal stress tolerance. Preserving NAD^+^ availability is proposed to support cellular defenses relevant to brain aging [12].

Epidemiological evidence reflects these mechanisms. In pooled analyses of over a million participants, higher midlife BMI is linked to greater long-term dementia risk [13]. Short follow-up studies can falsely show lower BMI before diagnosis because of prodromal weight loss. Thus, the timing of exposure matters. Midlife excess adiposity is harmful for later cognitive outcomes [14]. Dietary patterns rich in minimally processed plant foods, unsaturated fats, and marine omega-3s support healthier cognitive trajectories [15]. Randomized data from PREDIMED show better global cognitive test performance after long-term Mediterranean diet interventions with extra-virgin olive oil or nuts, compared with a low-fat control diet [16]. Observational studies on the MIND diet, a Mediterranean–DASH hybrid emphasizing leafy greens and berries link higher adherence to markedly lower Alzheimer’s disease incidence, even after multivariable adjustment [17]. Recent UK Biobank analyses report that Mediterranean diet adherence associates with reduced dementia risk independent of polygenic risk, highlighting the potential importance of diet quality even in genetically susceptible individuals [18].

**Figure 1:**
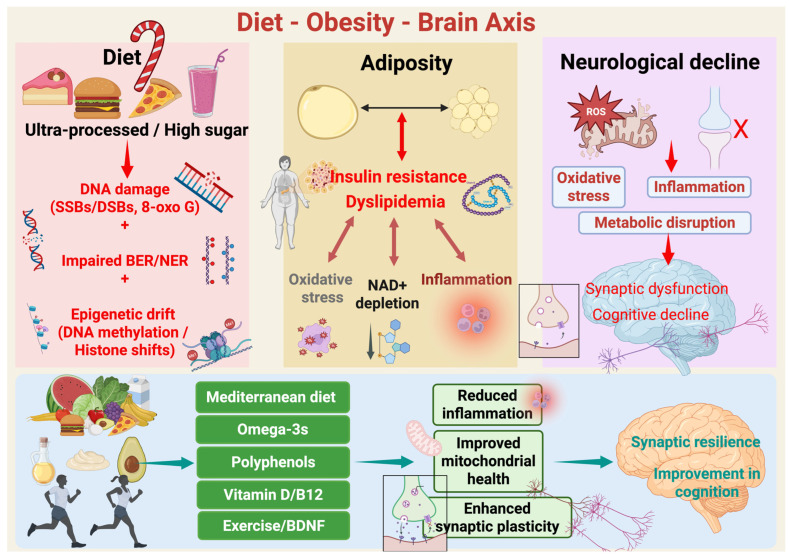
Diet–Obesity–Brain axis. Schematic overview of how diet quality modulates adiposity, systemic metabolism, and brain aging. Chronic consumption of ultra-processed, energy-dense, high-sugar foods is associated with genomic stress, including oxidative DNA lesions (e.g., single- and double-strand breaks and 8-oxoG), impaired efficiency of base excision and nucleotide excision repair pathways (BER/NER), and cumulative epigenetic drift at the level of DNA methylation and histone state. These diet-caused molecular damages arise concurrently with excess adiposity and metabolic derangement. Nevertheless, excess adiposity is marked by insulin resistance and dyslipidemia. These conditions enhance whole-body and CNS-pertinent oxidative stress, chronic low-grade inflammation, and NAD^+^ depletion, a critical metabolic cofactor essential for mitochondrial function, sirtuin activity, and DNA repair. Combined, these processes feed forward and sustain midlife obesity’s metabolic stress. Additionally, in the brain, the resultant oxidative stress, inflammation, and bioenergetic disruption induce microglial activation, mitochondrial reactive oxygen species (ROS) formation, and synaptic failure. With time this leads to impaired synaptic plasticity, compromised network integrity, and cognitive diminution. Finally, the protective levers such as Mediterranean-style diet patterns, omega-3 fatty acids, foods rich in polyphenols, and optimal vitamin D and B12 status, coupled with exercise and resultant increases in brain-derived neurotrophic factor (BDNF), reverse these mechanisms. These interventions reduce inflammation, enhance mitochondrial function, maintain NAD^+^-supported repair capacity, and augment synaptic plasticity, thus ensuring synaptic resilience and preservation of cognitive function over aging. Created in BioRender. Mishra, P. (2025) https://BioRender.com/nw9bkrz.

Multiple biological pathways likely mediate these protective effects. Polyphenols and monounsaturated fats reduce neuroinflammation and oxidative injury [19]. Omega-3 fatty acids influence membrane composition and balance inflammatory eicosanoids. Fiber-rich diets improve insulin sensitivity and foster a eubiotic gut microbiota [20]. This microbiota communicates with the brain through immune, endocrine, and vagal routes. The microbiota–gut–brain axis has emerged as a key channel by which diet shapes brain physiology and behavior across the lifespan [21]. Diet may also affect the pace of biological brain aging through epigenetic mechanisms. Population studies link healthier lifestyles including diet and physical activity to slower DNA methylation clocks [22]. Small randomized trials that combine diet improvements with exercise, sleep, and stress reduction show modest but measurable reductions in epigenetic age estimates [23]. Larger and longer trials are still needed. The clock changes should be interpreted with caution. Most diet–clock findings are associative; DNA methylation clocks are not validated surrogate endpoints for cognitive outcomes. Trials reporting clock changes should prespecify cognitive and mechanistic co-endpoints and avoid causal claims from clock deltas alone. Building on this foundation, the review will bring together key evidence. (i) It will examine the bidirectional links among diet, obesity, neuroinflammation, and oxidative stress. (ii) It will assess effects on DNA repair, NAD^+^/sirtuin signaling, and epigenetic aging. (iii) It will review evidence that Mediterranean and MIND diets protect cognitive health. (iv) It will highlight translational implications for prevention and intervention. The overall goal is to integrate mechanistic and clinical findings. The overall snapshot of the diet–obesity–brain axis can be explained as given in Figure 1. This article is a narrative review informed by a structured search of PubMed, Scopus, and Web of Science, with explicit attention to human evidence linking diet, adiposity, inflammation, DNA repair/NAD^+^ metabolism, epigenetic aging, and cognition; it is not a formal systematic review.

### Search Strategy and Selection Criteria

This review is a narrative synthesis informed by a structured literature search. We queried PubMed, Scopus, and Web of Science for articles published till October 2025 using combinations of terms covering (i) dietary patterns (Mediterranean, MIND, DASH, Nordic, traditional Asian, ultra-processed food); (ii) obesity, adiposity, insulin resistance, and systemic inflammation; (iii) oxidative and nitrosative stress, mitochondrial dysfunction, and blood–brain barrier integrity; (iv) DNA repair, PARP, sirtuins, and NAD^+^ metabolism; (v) epigenetic aging, DNA methylation clocks, chromatin state; (vi) cognition, neurodegeneration, dementia, and brain aging; and (vii) artificial intelligence, machine learning, digital phenotyping, and automated dietary assessment. We prioritized human observational cohorts, randomized and multidomain intervention trials, meta-analyses, and mechanistic reviews that directly linked diet, metabolic state, and brain-relevant outcomes. Landmark preclinical studies were included when they clarified a pathway that is already observed in humans (for example, gut barrier permeability and microglial activation, or NAD^+^/sirtuin–PARP signaling in neuronal stress responses). We excluded studies that did not address brain- or cognition-relevant outcomes, studies without enough methodological detail to interpret, and purely speculative opinion pieces. Reference lists of key reviews and clinical trials were hand-searched to identify additional relevant work. Because the goal is mechanistic integration and translational framing rather than formal effect-size pooling, this article does not claim PRISMA-level systematic review status. Instead, we explicitly distinguish associative evidence (e.g., prospective diet–cognition links) from interventional evidence (e.g., multidomain lifestyle trials), and we note where causality is not established.

## 2. Mechanistic Interfaces: How Diet and Obesity Stress the Brain Genome

Modern obesogenic diets high in saturated fats and refined sugars but low in micronutrients strain the brain through combined metabolic, inflammatory, and epigenetic pathways [24]. These factors work together to erode genomic maintenance in post-mitotic neurons and glia [25]. They also impair synaptic plasticity and speed cognitive decline [26]. Below, we outline five mechanistic interfaces where diet and adiposity converge on the brain’s genome and its protective systems.

### 2.1. The Pathway of Nutrient Excess via ROS Resulting in DNA Lesions in Long-Lived Neurons

Excess calories and lipid overload shift cellular redox set-points and increase mitochondrial reactive oxygen species (ROS) [27]. In the brain, ROS readily oxidize DNA bases such as 8-oxoG and create strand breaks [28]. Neurons, which are long-lived and largely non-dividing, must repair this damage continuously. When repair is incomplete or delayed, lesions accumulate. They trigger transcriptional noise, stall RNA Pol II, and activate cell-death pathways linked to neurodegeneration [6,9]. Adiposity worsens this redox stress through chronic metaflammation, a low-grade inflammatory state driven by nutrient sensing in adipose tissue and liver [29]. Inflammatory signals spill over into the central nervous system. Hypothalamic and extra-hypothalamic microglia become primed, increasing cytokine and nitric oxide production. These changes further damage nucleic acids [3,30,31].

### 2.2. The NAD^+^–Sirtuin–PARP Axis Under Caloric Excess

Genomic maintenance in neurons relies heavily on NAD^+^. NAD^+^ serves as a co-substrate for DNA repair enzymes (PARPs) and deacetylases (SIRT1/6) that maintain chromatin stability and stress responses [32]. In inflammatory and oxidative conditions, PARP-1 is hyper-activated by DNA breaks. This activity consumes NAD^+^ and limits sirtuin function. The result is a “NAD^+^ tug-of-war” that can suppress mitochondrial biogenesis and repair programs [12,33]. Conversely, inhibiting PARP or reducing PARP-1 activity raises cellular NAD^+^ and activates SIRT1. This shift improves oxidative metabolism. SIRT6 also works with PARP-1 to coordinate double-strand break repair, tightly linking NAD^+^ balance to genome integrity [33,34,35]. In humans, oral NAD^+^ precursors increase circulating NAD^+^. For example, nicotinamide riboside (NR) elevates blood NAD^+^ both acutely and chronically. Nicotinamide mononucleotide (NMN) also raises serum NAD(H) in randomized trials, though definitive CNS outcomes remain unproven [36,37].

### 2.3. One-Carbon Metabolism, Methyl Donors, and the Brain Methylome

DNA repair and chromatin regulation closely interact with one-carbon metabolism (folate–B_12_–choline). This pathway supplies S-adenosylmethionine (SAM), the universal methyl donor. Dietary folate and B_12_ status therefore shape DNA and histone methylation patterns in the brain [38]. These patterns influence neurodevelopmental gene expression, myelination, and synaptic function. Disruptions in this axis such as folate deficiency, B_12_ deficiency, or excess folate combined with low B_12_ can cause hyper- or hypomethylation at neural loci. They can also worsen oxidative damage by raising homocysteine levels [39,40]. Maternal B_12_ deficiency with relatively high folate has been linked to greater adiposity and insulin resistance in offspring. This developmental imprinting later affects brain insulin pathways and promotes neuroinflammation [41].

### 2.4. Inflammation, BBB Permeability, and the Gut–Brain Corridor

Obesity triggers neuroinflammation that spreads beyond the hypothalamus to the hippocampus and cortex [3,42]. In both rodents and humans, diet-induced obesity causes hypothalamic neuronal injury and gliosis. High-fat or Western diets also impair blood–brain barrier (BBB) integrity. This increases paracellular leak and allows circulating cytokines and lipopolysaccharide to enter the brain. These factors amplify DNA damage and the need for repair in neural cells [43,44]. The gut microbiota influences this interface [45]. Germ-free mice have higher BBB permeability than conventionally colonized controls [46]. This indicates that microbial signals help maintain BBB integrity. Obesogenic diets can disrupt this process through gut dysbiosis [46].

### 2.5. Neurotrophins and Activity-Dependent Genome Care

Diet and physical activity influence neurotrophin signaling, such as BDNF. BDNF links synaptic plasticity to transcriptional and chromatin programs that protect the genome [47]. Diets rich in polyphenols and omega-3s increase BDNF and antioxidant defenses. Excess saturated fat and sugar reduce them [1]. Exercise provides a complementary benefit. In overweight or obese adults, an 8-week exercise program restored brain insulin responsiveness. It also improved hippocampal network connectivity, offering a way to reverse diet-linked insulin resistance and genomic stress [48]. Obesogenic nutrition increases oxidative lesions in the brain, depletes NAD^+^ needed for DNA repair, and disrupts methyl-donor availability for chromatin. It also opens the BBB to inflammatory signals and reduces neurotrophin-coupled plasticity. All of these effects converge on the same outcome: reduced genomic maintenance in neurons and glia. These mechanisms point to multipronged interventions. Strategies include correcting dietary patterns to lower ROS and restore methyl donors, conserving or replenishing NAD^+^, supporting the microbiome to strengthen the BBB, and exercising [49]. Together, these approaches help relieve the genome from continuous metabolic stress.

## 3. Evidence by Dietary Exposures and Nutrients

### 3.1. Dietary Patterns Linked to Better Cognitive Outcomes

Long-term dietary patterns, rather than isolated nutrients, show the clearest relationship to trajectories of cognitive aging. Higher adherence to Mediterranean-style and MIND diets is consistently associated with slower global cognitive decline and lower risk of incident cognitive impairment in longitudinal cohorts. Also, the Mediterranean- or MIND-like food patterns have produced domain-specific improvements in memory and executive function in randomized settings, although most of the randomized evidence comes from multidomain lifestyle programs rather than diet alone [50,51]. Randomized multidomain lifestyle trials that include Mediterranean- or Nordic-style nutrition guidance report preservation of specific cognitive domains, particularly memory and executive function, in older adults with elevated vascular and metabolic risk [52,53]. These data support a mechanistic link among diet quality, adiposity and insulin resistance, systemic inflammation, vascular stress, and brain aging, but they do not establish that diet alone prevents dementia. The Dietary Approaches to Stop Hypertension (DASH) pattern, Nordic-style patterns rich in whole grains, berries, and rapeseed oil, and traditional predominantly plant- and fish-based Asian patterns show similar features (high intake of fiber-rich plant foods and unsaturated fats, lower intake of ultra-processed, energy-dense foods) and have been linked to better vascular/metabolic profiles and more favorable cognitive trajectories in aging populations [52]. In PREDIMED substudies and a dedicated randomized trial, a Mediterranean diet enriched with extra-virgin olive oil or mixed nuts improved composite cognition compared with a low-fat control. Benefits were seen over ~4 years in older adults at vascular risk. Domain-specific improvements in memory and executive function were observed after ~2 years. Proposed mechanisms include reduced neuroinflammation and oxidative stress from polyphenols and MUFAs, improved insulin signaling, vascular protection, and effects on amyloid/tau biology [54]. A UK Biobank analysis (*N* ≈ 60,000; mean follow-up 9.1 years) reported lower incident dementia with higher Mediterranean diet adherence. This effect was independent of polygenic risk. These findings support a primary-prevention role for dietary quality, even among genetically susceptible individuals [18]. Similar cardiometabolic and neuroprotective signatures are reported for other culturally grounded patterns. The DASH dietary pattern, originally developed for blood pressure control, lowers blood pressure and improves vascular/metabolic risk profiles in controlled feeding trials and has been linked to better cognitive performance and slower cognitive decline in observational studies of older adults [55,56]. Nordic dietary patterns, which emphasize whole grains, root vegetables, berries, pulses, and rapeseed (canola) oil, have been associated with improved lipid profiles, reduced systemic inflammation, and better insulin sensitivity in intervention studies in Northern European populations, and emerging cohort data suggest more favorable cognitive trajectories with higher adherence [52,57]. Traditional predominantly plant- and fish-based Asian dietary patterns, which include vegetables, legumes/soy, fermented foods, and marine n-3 fatty acids with relatively low intake of animal saturated fat and ultra-processed products, have been associated with lower cardiometabolic burden and lower odds of late-life cognitive impairment in East Asian cohorts [58,59]. Across these patterns, recurring features include high intake of polyphenol- and fiber-rich plant foods, unsaturated fatty acids, and micronutrients (including B vitamins relevant to homocysteine metabolism and omega-3 fatty acids relevant to membrane signaling), together with lower intake of refined sugars, sodium-rich ultra-processed foods, and industrial trans fats. These shared features plausibly converge on vascular integrity, insulin sensitivity, gut–brain inflammatory signaling, and oxidative stress, which are central to the diet–obesity–brain axis reviewed here.

Because most dementia and cognitive-decline outcomes in this literature are observational, these associations should not be interpreted as proof of dementia prevention. Current randomized data mainly support intermediate benefits, including blood pressure control, lipid and insulin sensitivity improvements, and preservation of specific cognitive domains within multidomain lifestyle programs. The unifying inference is mechanistic rather than causal: culturally distinct dietary patterns that increase plant-derived bioactives and unsaturated fats and decrease highly processed, energy-dense foods tend to align with metabolic, vascular, and inflammatory profiles that are more compatible with healthy brain aging [50,51,55,56].

The MIND diet, a hybrid of Mediterranean and DASH diets tailored to neurodegeneration, is associated with substantially lower Alzheimer’s disease incidence in observational cohorts. Higher adherence also correlates with slower cognitive decline. Meta-analytic evidence is converging across cohorts, although randomized trials remain limited [60]. Mechanistically, the MIND diet emphasizes neuro-supportive foods such as leafy greens, berries, nuts, legumes, whole grains, olive oil, fish, and poultry. It limits red and processed meats, butter, pastries, and fried/fast foods. This pattern targets lipid profiles, vascular health, oxidative stress, and potentially amyloid/tau pathways (see Table 1). [17,61,62]. Multidomain interventions that include dietary counseling, such as FINGER, preserve or improve cognition in at-risk older adults over 2 years. These findings suggest that diet synergizes with exercise, cognitive training, and vascular risk control [52].

### 3.2. Specific Nutrients and Bioactives: What Holds Up?

Omega-3 long-chain PUFAs (EPA/DHA). Prospective studies and recent reviews suggest that higher intake or status of n-3 PUFAs, especially DHA, is associated with lower risk of cognitive decline and dementia [63]. Supplementation trials show mixed results. Modest benefits are seen in mild cognitive impairment (MCI) or populations with low baseline DHA [64]. Results are generally null in established Alzheimer’s disease or DHA-replete populations. Effects are likely moderated by dose, duration, EPA:DHA ratio, baseline status, and vascular/metabolic comorbidities. Neurobiological rationale includes membrane fluidity, synaptogenesis, neuroinflammation resolution, and cerebrovascular benefits [65,66].

Polyphenol-rich foods. Mechanistic and human studies support cognitive effects for select polyphenol classes. In a randomized study, cocoa flavanols improved dentate gyrus function and pattern recognition memory in older adults. These effects align with hippocampal angiogenesis and neurogenesis models. An 18-month randomized trial of a bioavailable curcumin formulation enhanced memory and attention. It also reduced amyloid/tau PET signal in limbic regions in non-demented adults. Generalizability and bioavailability of formulations remain important caveats [67,68,69].

B-vitamins (folate, B12, B6) and homocysteine. Elevated homocysteine is a risk factor for brain atrophy and cognitive decline [70]. In MCI, VITACOG trials showed that high-dose, homocysteine-lowering B vitamins slowed whole-brain atrophy by ~30% overall and ~50% in those with higher baseline homocysteine. They also selectively protected AD-vulnerable gray matter regions over 2 years. These effects are likely upstream of cognition and appear dependent on elevated baseline homocysteine. Benefits may not extend to advanced AD. [71,72].

Vitamin D. Observational cohorts consistently link low 25(OH)D to higher risks of all-cause dementia and AD, with thresholds around <50 nmol/L. However, large randomized trials in generally replete populations have not shown clear cognitive benefits. This supports a “deficiency-correction rather than universal supplementation” approach [73,74,75,76].

### 3.3. Dietary Exposures Linked to Worse Brain Outcomes (and How Obesity Mediates Risk)

At the other end of the spectrum, ultra-processed foods (UPFs) and Western-style high-fat/high-sugar (HFS) diets are linked to faster cognitive decline and higher dementia risk [77]. Mechanisms likely include excess adiposity, insulin resistance, vascular injury, microbiome dysbiosis, and neuroinflammation. In a Brazilian cohort of 10,775 participants (median follow-up 8 years), higher UPF consumption was associated with faster global and executive function decline. UK Biobank data also show that higher UPF intake correlates with greater dementia risk. These findings align with mechanistic studies demonstrating hippocampal vulnerability to HFS diets [78,79]. Obesity may mediate part of the diet–brain risk. Chronic low-grade inflammation, impaired insulin/IGF-1 signaling, and cerebrovascular disease are key pathways. Meta-analyses and lifecourse studies consistently link midlife obesity to higher later-life dementia risk. Late-life associations are more complex, partly due to confounding by prodromal weight loss. Dietary strategies that improve adiposity and metabolic health such as Mediterranean or MIND diets, higher fiber and polyphenols, and lower UPF and refined sugar intake may protect the brain indirectly. They act on both neural and cardiometabolic pathways [78,80].

**Table 1:** Snapshot of dietary exposures, mechanisms, obesity links, and representative human evidence.

Dietary Exposure	Key Neurobiological Mechanisms	Obesity/Metabolic Interaction	Representative Human Evidence	Practical Intake Cues
Mediterranean diet (EVOO, nuts, fish, whole grains, produce)	↓Neuroinflammation/oxidative stress (polyphenols, MUFAs), ↑endothelial NO and perfusion, ↑synaptic plasticity	Improves lipids, insulin sensitivity, BP—reduces obesity-mediated neurovascular risk	[6,9]	≥5–7 servings plants/day; olive oil as main fat; nuts most days; fish 2×/week
MIND diet	Emphasizes leafy greens, berries; limits saturated fats/processed foods; targets amyloid/tau, vascular, oxidative stress pathways	Lower SFA/UPF reduces adiposity and systemic inflammation	[17]	Leafy greens daily; berries ≥2×/week; nuts, legumes, whole grains; minimal fried/fast foods
Omega-3 (EPA/DHA)	Membrane fluidity, synaptogenesis, pro-resolving mediators, anti-thrombotic	Improves TGs and vascular risk; may modulate adiposity inflammation	[66]	Oily fish 1–2×/week or tailored DHA/EPA if low status
Polyphenols (e.g., cocoa flavanols; bioavailable curcumin)	↑Neurotrophins, angiogenesis, antioxidant defenses; ↓amyloid/tau aggregation and neuroinflammation	Often co-occur with high-fiber foods improving glycemic control	[69]	Dark cocoa (high-flavanol), berries, herbs/spices (bioavailable forms where studied)
B-vitamins (folate, B12, B6)	↓Homocysteine; methylation pathways; white-matter integrity	Links to insulin resistance and vascular health in deficiency states	[72]	Screen and correct deficiency/high tHcy rather than blanket supplementation
Vitamin D	Neurosteroid actions; immune modulation; vascular health	Low 25(OH)D common with obesity; adipose sequestration	[76]	Target sufficiency (e.g., 50–75 nmol/L) by sunlight, food, or supplements if deficient
UPF/Western HFS	↑Neuroinflammation, impaired insulin signaling; hippocampal vulnerability; microbiome dysbiosis	Drives weight gain, visceral adiposity, dyslipidemia	[78]	Replace UPF with minimally processed, fiber-rich foods; cap added sugars/saturated fats

Across RCTs and cohort studies, higher dietary quality (Mediterranean/MIND) is consistently associated with better cognitive trajectories. Mechanisms include effects on synaptic function, inflammation, vascular health, and amyloid/tau biology [81]. Targeted nutrients such as DHA, EPA, polyphenols, and B-vitamins show context-dependent benefits. These benefits are strongest when baseline biology is unfavorable, such as low DHA or high homocysteine. They also depend on delivering nutrients in bioavailable forms for sufficient duration. In contrast, UPF-heavy and high-fat, high-sugar diets impair hippocampal function. They worsen obesity-related metabolic injury and accelerate cognitive decline. Together, these findings support a food-first strategy. This approach reduces adiposity and modulates neurobiological pathways. Deficiency testing for B12, 25(OH)D, omega-3 index, and homocysteine can guide supplementation when needed.

## 4. The Microbiome–Gut–Brain Axis: How Diet and Obesity Transmit Neuroepigenomic Stress

Diet rarely acts directly on neurons. Instead, it reshapes the gut ecosystem, which in turn transmits immune, endocrine, metabolic, and neural signals to the brain. The gut can shift quickly even a few days of macronutrient changes reshape the microbiome. Over the long term, dietary patterns matter even more. A Western high-fat, high-sugar diet pushes the microbiome one way. A Mediterranean-like diet pushes it another. When obesity develops, the gut wall becomes leaky. Bacterial products like LPS leak into the blood. This creates low-grade endotoxemia. Endotoxemia sparks chronic, body-wide inflammation. That inflammation not stay in the body it reaches the brain [82]. Signals travel through the immune system, hormones, metabolites, and even the vagus nerve. Once inside the brain, these signals add stress. They challenge the brain defenses, including oxidative stress responses, DNA repair, and chromatin regulation [83].

### 4.1. The Barrier Interface: From Gut Leak to BBB Leak

In germ-free mice, the blood–brain barrier (BBB) is leaky. They have fewer tight-junction proteins holding the barrier together. When the gut is colonized, or when short-chain fatty acids (SCFAs) are added, the BBB becomes stronger. This shows that gut microbes help keep the brain’s barrier intact [84]. In obesity or chronic inflammation, things look different. LPS and cytokines slip through and weaken the BBB [85]. Loss of BBB integrity increases CNS exposure to circulating cytokines and endotoxin. This exposure elevates oxidative and nitrosative stress and increases DNA damage signaling in neural cells, thereby increasing repair demand. Conceptually, diet-induced gut dysbiosis can promote intestinal permeability and endotoxemia, which aggravate BBB dysfunction and microglial activation, linking peripheral inflammation to genomic stress in the brain.

### 4.2. Metabolite Signaling

Microbial metabolites can shape how the brain works [86]. Short-chain fatty acids (SCFAs) are a good example. Among them, butyrate stands out. It not only fuels gut cells but also acts as a signal. In the body, it works like a switch: it can inhibit enzymes called histone deacetylases (HDACs). It also activates receptors on cell surfaces, known as GPCRs. Through these actions, butyrate links gut microbes to brain biology. This links dietary fiber intake to chromatin remodeling and anti-inflammatory gene programs [87]. Preclinical studies show that SCFAs regulate microglial maturation. Butyrate and other SCFAs can also modify histone crotonylation and acetylation [88]. This provides a pathway from diet and microbes to the neural epigenetic state. Context is important: in an amyloid-prone mouse model, SCFA repletion increased Aβ deposition [89]. This highlights that timing, dose, and disease stage influence benefit versus risk. Lipopolysaccharide (LPS) and other microbe-associated signals activate innate immunity, including microglia, via TLR pathways. Sustained activation promotes neuroinflammation and can destabilize the BBB [90]. Conversely, the vagus nerve offers a rapid, bidirectional conduit. Gut signals, including microbial metabolites, can modulate afferent firing and central inflammatory reflexes. This provides a mechanistic framework for how diet and the microbiome influence mood and cognition [91].

### 4.3. Human Levers: Pattern, Fiber, Fermented Foods and Early Microbiome Trials

In older adults across five European countries, a 12-month Mediterranean diet shifted the gut microbiome toward taxa associated with lower frailty and inflammation. These changes suggest an inflammatory tone reaching the brain [92]. In healthy adults, a randomized diet trial showed that fermented foods such as yogurt, kefir, and kimchi increased microbiome diversity and lowered circulating inflammatory markers. Eating more fiber boosts the gut ability to break down carbohydrates. Over six weeks, this effect was noticeable but modest across the whole group. Still, the study shows that diet quality matters. Fiber and fermented foods are practical ways to shape the gut microbiome [93]. By doing so, they help set the body immune balance.

Researchers are exploring whether changing the gut microbiome can improve thinking and memory. In people with mild cognitive impairment (MCI), small trials have tested Lactobacillus plantarum C29-fermented soy and Bifidobacterium breve A1. One study even found higher levels of serum BDNF, a protein that supports brain health. Meta-analyses suggest the benefits are clearest in MCI, while results in Alzheimer’s disease are more variable [94]. This highlights a window for early intervention. These findings are still preliminary and depend on the right strain, dose, and duration. Mechanistic studies show that microbial products influence brain inflammation, blood vessel function, and gene regulation. The gut–brain axis acts as a bridge, sending signals from diet and body fat to the brain. Westernized diets and obesity can disrupt this system, leading to an unhealthy microbiome, leaky barriers, endotoxemia, and microglial activation. On the other hand, minimally processed diets rich in fiber, polyphenols, and fermented foods create a healthier metabolite environment. These include SCFAs, indole derivatives, and bile acids. They help protect the blood–brain barrier, maintain microglial balance, and support proper gene regulation in the brain [95]. A practical approach to neuroprotection could follow three steps: (1) improve dietary patterns with Mediterranean or MIND-style diets and reduce ultra-processed foods [96]; (2) support the microbiome with adequate fiber and fermented foods; and (3) explore targeted interventions, like strain-specific probiotics or synbiotics, focusing on early stages of disease or people at higher metabolic risk.

## 5. Translational Agenda and Outlook

**Figure 2:**
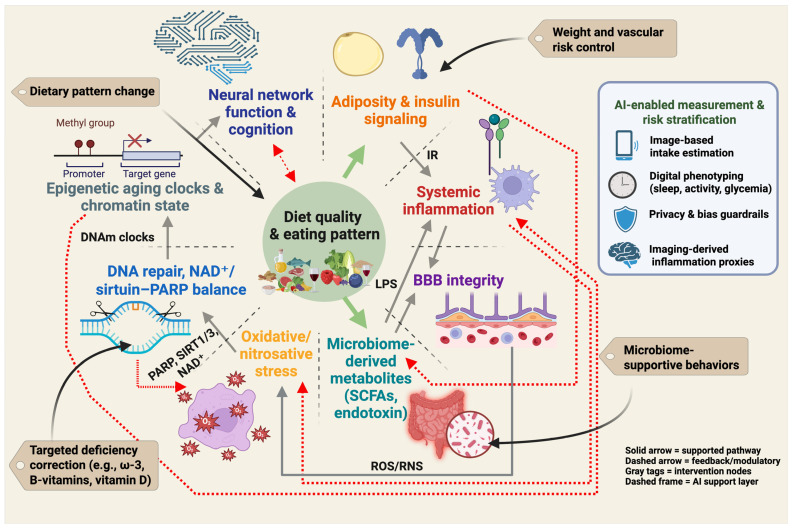
**Pattern-first prevention framework for the diet–obesity–brain axis.** Diet quality and overall eating pattern influence adiposity and insulin signaling, systemic inflammation, gut-derived metabolites and intestinal permeability, blood–brain barrier integrity, oxidative and nitrosative stress, DNA repair and NAD^+^/sirtuin–PARP balance, and epigenetic aging. These interconnected processes affect neural network function and cognition. Solid links indicate core pathways supported by human and mechanistic evidence. Dashed links indicate feedback and bidirectional modulation, including microbiome–inflammation signaling, reciprocal redox and repair load, and cognition shaping future dietary adherence. Gray callouts indicate intervention targets (dietary pattern change, weight and vascular risk control, microbiome-supportive behaviors, and targeted correction of specific deficiencies). The AI-enabled measurement and risk stratification panel illustrates current tools (image-based intake estimation, wearable-based digital phenotyping, privacy and bias guardrails, imaging-derived inflammation proxies) that can support exposure assessment and personalized monitoring. Created in BioRender. Mishra, P. (2025) https://BioRender.com/2nvp3a5.

Neuroprotective nutrition cannot be reduced to a single nutrient or a single biomarker. Instead, it operates as an interacting system in which dietary pattern quality influences adiposity and insulin signaling, systemic inflammation, gut-derived metabolites, blood–brain barrier function, redox stress, DNA repair and NAD^+^/sirtuin–PARP balance, epigenetic aging, and ultimately neural network integrity and cognition [32,97]. This systems view defines prevention targets at several levels: dietary pattern modification, weight and vascular risk management, microbiome-supportive behaviors, and correction of specific deficiencies. It also defines measurement targets for trials and clinical translation, including adiposity, inflammatory tone, barrier integrity, repair capacity, and biological age [52,98,99]. Figure 2 summarizes this pattern-first framework and indicates where emerging AI-enabled phenotyping may help quantify exposure, stratify risk, and monitor response.

Putting the evidence into practice supports a pattern-first approach, where diet is part of a broader prevention strategy. Both randomized trials and long-term cohort studies highlight Mediterranean-like and MIND diets as practical ways to protect cognitive health. In older adults with vascular risk factors, long-term trials show that a Mediterranean diet enriched with extra-virgin olive oil or nuts slows age-related cognitive decline. This approach outperforms simpler low-fat diets in preserving thinking and memory over time [54]. Large prospective cohorts indicate that higher Mediterranean adherence is linked to lower incident dementia, even after adjusting for polygenic risk [18]. Multidomain trials such as FINGER demonstrate that combined diet, exercise, cognitive training, and vascular risk management preserve or improve cognition over two years in at-risk older adults [52].

The diet–obesity–brain axis spans behavioral intake, adiposity and insulin signaling. It also includes the immune tone, BBB integrity, microbiome outputs, DNA repair capacity and neural network function. Artificial intelligence (AI) and machine learning (ML) enable (i) high-fidelity measurement of diet and physiology, (ii) prediction of cognitive trajectories under metabolic stress, and (iii) personalization of multidomain care [100]. On the measurement and phenotyping front, computer-vision models classify foods and portion sizes from images and wearable streams, reducing recall bias in dietary assessment, while ML applied to structural/functional MRI and diffusion data detects microstructural and connectivity patterns linked to obesity, insulin resistance, neuroinflammation, and oxidative-stress burden, complementing clinical scales. For mechanism mapping, multimodal learning can integrate NAD^+^/sirtuin/PARP proxies, homocysteine and one-carbon markers, inflammatory panels, microbiome-derived metabolites (e.g., SCFAs), and epigenetic clocks to infer pathway activity and interactions (e.g., PARP-driven NAD^+^ drain vs. sirtuin tone) that may be invisible to single-omic analyses. In risk stratification and treatment targeting, predictive models can identify phenotypes most likely to benefit from Mediterranean/MIND dietary patterns, weight reduction, homocysteine-lowering B-vitamins, omega-3 optimization, fermented foods, or emerging NAD^+^-supportive adjuncts, prioritizing midlife adults with adiposity and metabolic syndrome. For trial design and monitoring, AI can support adaptive lifestyle trials by forecasting adherence, automatically quantifying diet quality (ultra-processed food exposure, fiber intake, polyphenol proxies), and tracking brain-relevant endpoints (cognitive composites, imaging signatures) to accelerate learning cycles [101]. Finally, robust guardrails are essential: to avoid bias and overfitting, models should be developed on diverse cohorts, report calibration across sex, age, and metabolic strata, pre-register analysis plans, and incorporate privacy-preserving pipelines for images and wearable data; clinical use should emphasize model explanation aligned with known biology (e.g., NAD^+^/repair, neuroinflammation, BBB integrity) rather than black-box scores alone.

Concrete applications include (i) computer-vision estimation of foods and portion sizes from meal images to reduce recall bias; (ii) digital phenotyping from wearables to quantify sleep, activity, and glycemic variability that modulate brain-relevant metabolism; (iii) MRI- and diffusion-based models that detect patterns related to neuroinflammation and adiposity; and (iv) machine-learning classifiers that score the degree of food processing for exposure quantification. Limitations include algorithmic bias across sex, age, and metabolic strata; privacy risks from images and accelerometry; and limited interpretability. Clinical deployment should use diverse training sets, calibration reporting, privacy-preserving pipelines, and explanations aligned with known biology rather than black-box risk scores. 

Several AI applications already map onto the diet–obesity–brain axis described in this review. First, image-based dietary assessment systems use computer vision to identify foods and estimate portion size and nutrient content from meal photographs or wearable cameras, which reduces the recall bias and under-reporting that limit traditional food diaries. Deep convolutional models can segment mixed dishes, infer volume, and generate energy and macronutrient estimates in near real time, and egocentric wearable-camera pipelines have been field-tested across demographically distinct cohorts, including non-Western populations, showing feasibility for objective intake capture outside of controlled clinics [102]. These vision systems are being positioned as scalable intake monitors for obesity, diabetes, and cardiometabolic risk management [103,104]. Second, multimodal ‘digital phenotyping’ links continuous wearable data (sleep regularity, movement patterns, heart rate variability, glucose excursions from continuous glucose monitoring) with dietary logs to infer individual metabolic stress profiles relevant to brain aging. This approach is now used in precision nutrition programs that adapt meal timing and composition to an individual’s glycemic responses and circadian state, and it is being evaluated in metabolic and cognitive risk groups rather than only in healthy volunteers [105,106,107,108]. Third, machine learning applied to MRI and diffusion-based neuroimaging can identify obesity-associated neuroinflammatory signatures in hypothalamic and white-matter regions that regulate feeding, reward, and executive control, suggesting a possible noninvasive brain biomarker of metabolic injury, although these imaging markers remain indirect and require histopathologic anchoring before they can serve as endpoints [109,110,111,112]. These same pipelines are also being trained to classify the degree of food processing and to link ultra-processed dietary exposure with downstream inflammatory and vascular phenotypes, supporting mechanistic stratification in trials of dietary pattern modification [113]. However, AI-assisted nutrition raises unresolved translational constraints: model performance can degrade in under-represented groups (for example by sex, age, ancestry, disability status, or socioeconomic context), which risks biased risk scores and unequal recommendations; large-scale food images, CGM traces, and location-linked behavioral streams create substantial privacy and governance liabilities; and most high-performing models remain ‘black boxes,’ limiting clinical interpretability and regulatory acceptability [114,115,116,117]. A practical path forward is equity-aware personalized nutrition: algorithms trained and calibrated on diverse populations, explicit reporting of error by subgroup, privacy-preserving pipelines for diet and wearable data capture, and biological interpretability that ties AI outputs to known mechanisms such as adiposity, inflammatory tone, NAD^+^/sirtuin–PARP repair capacity, and vascular–barrier integrity rather than opaque composite scores [114,115,116].

These results reinforce that dietary changes are most effective when paired with complementary lifestyle interventions. Because midlife adiposity amplifies neuroinflammation, vascular injury, and insulin resistance, weight and metabolic control are key translational targets. Practically, this involves prioritizing minimally processed, plant-forward foods. Refined starches, added sugars, and saturated fats should be replaced with whole grains, legumes, unsaturated oils, and marine fish. Ultra-processed foods (UPFs) should be reduced, as they are linked to faster cognitive decline and higher dementia risk [78]. These recommendations align with consensus prevention frameworks emphasizing modifiable lifestyle risks across the life course [118].

A targeted screen-and-correct approach to micronutrients complements dietary pattern changes. Elevated plasma homocysteine is a modifiable risk marker for neurodegeneration. In mild cognitive impairment, B-vitamin therapy (folate, B12, B6) slowed whole-brain atrophy and protected Alzheimer’s vulnerable gray matter, with the greatest benefits in individuals with high baseline homocysteine [72,119]. For vitamin D, observational studies link deficiency to higher dementia risk. Trials in generally replete populations are largely neutral. This supports a deficiency-correction rather than universal supplementation approach [76,120]. Assessing long-chain omega-3 status, such as the red-blood-cell Omega-3 Index, can guide personalized intake of dietary fish or supplements in low-status individuals. However, dementia-specific thresholds are not yet established [121]. Upstream immune tone can be influenced through microbiome-supportive nutrition. A controlled feeding trial showed that increasing fermented foods, including yogurt, kefir, and kimchi, enhanced microbial diversity and lowered circulating inflammatory markers [122]. This provides a practical route to dampen systemic inflammation, which supports brain health. Pharmacologic or nutraceutical adjuncts remain investigational.

Nicotinamide riboside and β-nicotinamide mononucleotide reliably raise circulating NAD^+^ in humans. Durable cognitive benefits and CNS target engagement are not yet established. These agents should be evaluated within lifestyle-anchored trials rather than used as first-line, stand-alone strategies [36,37]. Future implementation can be improved by risk stratification, focusing intensive programs on midlife adults with obesity, metabolic syndrome, or high homocysteine. Embedding mechanism-proximal endpoints in lifestyle trials such as homocysteine and omega-3 status, inflammatory panels, and cautiously interpreted epigenetic-clock readouts may capture diet-responsive aging biology [22,23]. Overall, a staged plan offers the clearest evidence-aligned pathway for protecting brain health: (i) prioritize diet quality as the foundation; (ii) maintain weight and metabolic control; (iii) correct targeted deficiencies; (iv) support the microbiome through diet.

## 6. Conclusions

Diet influences brain aging through interacting metabolic and molecular pathways. Patterns that promote excess adiposity and insulin resistance amplify systemic inflammation and oxidative/nitrosative stress, erode DNA-repair capacity and NAD^+^/sirtuin–PARP balance, and impair barrier function along the gut–brain axis. These processes converge on synaptic integrity, network efficiency, and cognitive performance. In contrast, Mediterranean- and MIND-style dietary patterns are associated in prospective cohorts with slower cognitive aging, and randomized multidomain lifestyle trials that include Mediterranean- or Nordic-style nutrition guidance report preservation of specific cognitive domains in older adults at elevated vascular and metabolic risk. These data support a mechanistic link among diet quality, adiposity, vascular stress, and neuroinflammation, but they do not yet prove dementia prevention by diet alone. Diets high in ultra-processed, high-fat/high-sugar foods align with the opposite profile: greater adiposity, dysbiosis, barrier dysfunction, and higher inflammatory load. AI-enabled nutrition technologies can assist exposure measurement (for example, image-based intake estimation), metabolic phenotyping from wearables (sleep, activity, glycemic variability), and individualized risk stratification, provided they are developed and deployed with privacy, bias, and interpretability safeguards and are used to support mechanism-guided lifestyle interventions rather than to generate opaque risk scores. From a translational perspective, the clearest path forward is a staged, pattern-first strategy. This strategy prioritizes weight and metabolic control. It screens and corrects common deficiencies, including homocysteine/B-vitamins, 25(OH)D, and, when indicated, omega-3 status. It also incorporates microbiome-supportive practices emphasizing fiber and fermented foods. Pharmacologic or nutraceutical adjuncts, such as NAD^+^ precursors, remain promising but unproven for durable cognitive outcomes. These agents should be evaluated within lifestyle-anchored trials rather than used as stand-alone interventions. Future research should focus on improved risk stratification, particularly at midlife. It should embed mechanistic biomarkers, including metabolic and inflammatory panels, homocysteine, and omega-3 status. Cautiously interpreted epigenetic clocks and microbiome readouts can help link dietary change with brain-relevant biology. Together, these steps provide a pragmatic, evidence-aligned framework. This framework is intended to lower diet-related brain risk in a way that is biologically informed, testable in trials, and ethically monitorable.

## Data Availability

No new data were created or analyzed in this study. Data sharing is not applicable to this article.

## Abbreviations

The following abbreviations are used in this manuscript:

AD	Alzheimer’s disease
BBB	Blood–brain barrier
BDNF	Brain-derived neurotrophic factor
BER	Base excision repair
CNS	Central nervous system
DASH	Dietary Approaches to Stop Hypertension
DHA	Docosahexaenoic acid
DSB	Double-strand break
EPA	Eicosapentaenoic acid
HDAC	Histone deacetylase
LPS	Lipopolysaccharide
MCI	Mild cognitive impairment
MIND	Mediterranean–DASH Intervention for Neurodegenerative Delay
MUFA	Monounsaturated fatty acid
NAD^+^	Nicotinamide adenine dinucleotide
NER	Nucleotide excision repair
PARP	Poly(ADP-ribose) polymerase
PET	Positron emission tomography
PUFA	Polyunsaturated fatty acid
RCT	Randomized controlled trial
ROS	Reactive oxygen species
SAM	S-adenosylmethionine
SCFA	Short-chain fatty acid
tHcy	Total homocysteine
UPF	Ultra-processed food(s)
